# Major Determinants of Innovation Performance in the Context of Healthcare Sector

**DOI:** 10.3390/ijerph20065007

**Published:** 2023-03-12

**Authors:** Abdelmohsen A. Nassani, Asad Javed, Joanna Rosak-Szyrocka, Ladislav Pilar, Zahid Yousaf, Mohamed Haffar

**Affiliations:** 1 Department of Management, College of Business Administration, King Saud University, P.O. Box. 71115, Riyadh 11587, Saudi Arabia; 2 Department of Management Sciences, Hazara University, Mansehra 21120, Pakistan; 3 Department of Production Engineering and Safety, Faculty of Management, Czestochowa University of Technology, 42-200 Czestochowa, Poland; 4 Department of Management, Faculty of Economics and Management, Czech University of Life Sciences Prague, 16500 Prague, Czech Republic; 5 Higher Education Department, Government College of Management Sciences, Mansehra 21300, Pakistan; 6 Department of Management, Birmingham Business School, University of Birmingham, Birmingham B15 2TY, UK

**Keywords:** innovation performance, healthcare industry, innovation network, artificial intelligence adoption, digital innovation

## Abstract

Through the innovation network (IN) and the use of artificial intelligence (AI), this study aims to look into the innovation performance (IP) of the healthcare industry. Digital innovation (DI) is also tested as a mediator. For the collection of data, cross-sectional methods and quantitative research designs were used. To test the study hypotheses, the SEM technique and multiple regression technique were used. Results reveal that AI and the innovation network support the attainment of innovation performance. The finding demonstrates that the relationship between INs and IP links and AI adoption and IP links is mediated through DI. The healthcare industry plays a vital role in facilitating public health and improving the living standards of the people. This sector’s growth and development are largely dependent on its innovativeness. This study highlights the major determinants of IP in the healthcare industry in terms of IN and AI adoption. This study adds to the literature’s knowledge via an innovative proposal in which the mediation role of DI among IN-IP and AI adoption-innovation links is investigated.

## 1. Introduction

In the modern digital market, the conception of the innovation performance (IP) has been greatly acknowledged by advanced innovation scholars due to its competitiveness for all kinds of business sectors [1]. To achieve IP, businesses rely heavily on the continuous and significant function of artificial intelligence (AI) and the innovation network (IN). Furthermore, digital innovation (DI) is well known for the development of state-of-the-art techniques for performing economic procedures using novel digital web expertise to achieve IP objectives [2]. The internet and DI systems are viewed as a rapidly rising phenomenon for trades to boost the IP [2,3] of different industries, including the healthcare industry, through the use of AI and IN. The extensive use of DI technologies in business concerns, product development processes, and marketing strategies has significantly transformed big-business prototypes in various industries [1], like the healthcare industry. Improved IP achievement for the healthcare industry is a multifaceted and difficult task, and there is also a pressing need to incorporate AI and establish a strong IN [1]. 

Previous research shows that only a few studies on the healthcare industry have been conducted to investigate the preconditions for the growth of the IP [4]. The prior studies suggested that various attributes are results of the AI and INs and, at the same time, positively impact IP. The key outcome of both AI and IN is DI, which successively affects IP. Due to this significant gap, we examined how DI can bridge the connections between AI, IN, and IP. According to our present research, both AI and INs enable organizations to adapt executive resources to emerging technology, which ultimately results in an improvement in IP. This study model is unique and based on three diverse variables, i.e., IN and AI adoption as independent variables and DI as a mediator between IN, AI adoption, and IP links, and empirically tested their impact and outcomes on improved IP in the healthcare industry. Several researchers have overlooked significant factors that have sped up and improved the IP in the healthcare industry. However, to the best of our knowledge, studies have yet to be carried out with these study variables. To fill this gap, our current research aims to identify the relationship between IP, INs, and AI and examine the mediation effect of DI between them. This study empirically examines the impact of AI adoption and the IN on IP from the perspective of the digital market.

The digital nation submits to entire economic operations that depend on digitized information, expertise, and the latest technology-related knowledge [5]. The digital market has numerous outcomes that affect the performance of business firms [3], specifically the tourism industry. In the 21st century, business temperaments around the globe have changed due to the emergence of the most up-to-date digital technologies [2]. Service delivery with cutting-edge digital technologies requires AI and an IN for the accomplishment of innovative business prototypes [5]. AI refers to drastically changing procedures and the end products of the IP due to its specific nature and ontology [3]. The change engendered by means of AI is frequently diverse, as are those alterations triggered by customary information technology, as it is increasing the latest methods to practice and gather a massive amount of the latest innovative information [2]. AI has become the primary component of new industry models that focus on the achievement of DI and IP in firms [3].The IN accelerates the innovation process through organization and the use of HR and the most recent internet advancements [6]. According to numerous academics, businesses face a slew of significant challenges in managing and adapting to digital change as a result of a lack of innovative capabilities and competencies. The IN ensures the incorporation and modification of digital technologies in order to improve DI and IP in businesses [5]. Hence, AI and the IN perform a critical role in the achievement of DI toward new business prototypes. Furthermore, AI and INs help in suggesting the right path to achieve IP, and this method is further reliable with DI [7,8].

The paper arrangement is set in the following way. Section 1 includes a literature survey focusing on AI, INs, and IP. The methodology is the next section of the paper, followed by data analysis. The last section includes a discussion, implication, conclusion, limitations and future directions.

## 2. Literature Review

Existing literature on variables of current research is provided as under:

### 2.1. Artificial Intelligence and Innovation Performance

AI refers to the course of action of human intelligence through machines similar to computer systems and particular applications like natural language processing, machine versions, expert systems, etc. [3,8]. It helps acquire valuable productive information, forecast and adopt market trends, and enhance IP [9]. The healthcare industry involves businesses that manufacture drugs and medical equipment and also offer supply, delivery, and clinical services [4]. AI is the capability of computers to control robots to do jobs commonly done by intelligent beings [10]. This study presents a close association between AI and IP. AI is rising as a new source in the digital economy for achieving innovation performance [11]. AI makes it easier for an organization to share information and knowledge by making it easier to find IP [12]. AI allocates firms to use external and internal resources of the enterprise and adopt rapid changes in the market for the development of new products, which leads to improvements in IP [13]. AI helps an organization obtain new ideas, develop new products, redesign existing ones, and act as an efficient means of improving IP [14]. Our research supports previous studies about the fact that AI plays a significant role as part of traditional firm planning strategies that assist firms in effectively dealing with emerging opportunities and raise challenges for developing new products that enhance IP in the digital economy [15]. Therefore, AI has the capability to activate innovation in the firm. AI increases innovation processes as an internal aptitude of the firm and acts as an inventive response to challenges and opportunities, increasing the IP of the firm. It helps in the development and research processes by exploiting new information and knowledge and capturing opportunities using innovative ways that help improve innovation performance [13].

**Hypothesis 1.**  *AI is positively linked with the innovation performance of the healthcare industry.*

### 2.2. Innovation Network and Innovation Performance

The IN is comprised of the informal and official connections between businesses, which they can utilize to obtain implicit knowledge, reports, software, and system knowledge to increase their IP [16]. Organizations find it useful to integrate INs to find the resources, knowledge, and concepts they need to improve their IP [17]. The usage, absorption, and integration of knowledge into firms will be influenced by the structural characteristics of an IN, which will then have an impact on the firm’s IP [18]. Firms can exchange and obtain a vast sum of implicit knowledge via an IN through the flexibility of the speedy changes that are critical for enhancing IP [19]. The IN facilitates firms’ adoption of advanced innovative competencies by gathering new knowledge, resulting in improved IP [20]. It provides organizations with a platform for inventive information acquisition and learning about quick changes to increase the IP of the firm [12].

**Hypothesis 2.**  *There is a positive relationship between the innovation network and innovation performance.*

### 2.3. Artificial Intelligence and Digital Innovation

Artificial intelligence (AI) refers to the capability to stay connected to the world of business through communication and e-marketplaces, which help in low-cost scaling and prompt services [12]. It is the science of creating intelligent machines that can help humans to perform tasks faster, more efficiently, and better than before [3]. AI opens up previously untapped infinite possibilities and opportunities, facilitating society’s use of advanced technologies in day-to-day activities, which leads to DI [14]. AI is the ability to stay in touch with the business world through communication and e-marketplaces, which help with cost-effective scaling and quick service. It transforms society through emerging technologies to further digitalize it through the implementation of new technologies in the numerous activities that were unknown until that time through DI [15]. DI provides new advanced services and inputs and uses AI to improve the outcomes of these inputs, which play an important role in firm performance [2]. AI proposed novel approaches for information processing that is used to generate the most recent waves of DI [3]. ’DI’ is new digital knowledge that will improve the application and facilities of future policies and proposals [21]. AI enables physical and digital components to produce new market offerings, services, and products, generate new business models and processes, and increase the DI process [2]. AI promotes DI in firms through building, reconfiguring, and integrating external and internal abilities to deal with a rapidly changing environment [21]. AI can formulate sense from knowledge and information gathered from past experiences, which supports dealing with the uncertainty of future actions [22]. DI engages in the formation of new operating ways for firms through the support of AI, which consequently influences how transactions are digitally performed, decisions are taken, and work is done [15]. AI is increasingly used in various organizations to accelerate and support information processes such as detailed information in numerous interviews and resumes; thus, AI features, such as recommendation, storage, and analysis, enable new approaches that support information processing, which contribute to DI [14].

**Hypothesis 3.**  *AI is positively linked with digital innovation.*

### 2.4. Innovation Networks and Digital Innovation

To achieve success through DI through digital technology, innovation artists must join and establish INs based on various knowledge, resources, and competencies [23]. The IN is a representation of the modular industry arrangement that lets self-regulating companies bring DI to the e-market [24].The IN is based on the new DI technology, which is divided into four layers: the service layer, the network layer, the content layer, and the device layer [6].Designers use standardized interfaces to combine different layer components to create DI [25]. The IN permits the best actors within each layer to make innovative products and services because consumers want DI from a match or mix of innovative components [25]. The incorporation of communication technology and digital computers that traditionally convert non-digital services and products to digitalized versions [26,27] is referred to as DI. An IN needs different performers from diverse fields to work together; this heterogeneity of knowledge acts as the base for DI [28]. INs facilitate users’ use of computing capabilities designed for organizational work and create interactions among users with advanced digital technology, with growing recognition of DI [29]. The IN fosters a desire for collaboration across organizational boundaries [30]. Through DI, INs provide access to various novel information resources and capabilities, as well as interaction among artists, which boosts innovativeness in firms [31].

**Hypothesis 4.**  *Innovation network predicts digital innovation.*

### 2.5. Digital Innovation and Innovation Performance

DI enables organizations to collect and share information and knowledge, which improve IP and allows for new e-market trends [32]. DI enables organizations to collect and share information and knowledge, which improves IP and helps them get used to new e-market trends [32]. It increases organizations’ internal innovation advantages through product improvement and new product development processes that lead to improved IP. DI can boost an organization’s competence in designing and developing new products and enhance its IP [9]. DI enhances a firm’s capability to share and obtain knowledge and information in the databases that help design new product features and improves the quality of the innovative product [32]. It shortens the development phase, reduces development costs, and aids in the completion of new artifact designs, resulting in good IP for the firm [1].It strengthens the firm’s capability to commercialize its new design product.

Seeking e-market knowledge and trends to aid in the introduction of new products into the marketplace to meet consumers’ rapidly changing needs and improve IP [33]. DI does not automatically produce higher IP at the organizational level until it seeks knowledge about the e-market innovation process and collects and shares it with the firm [34]. To attain IP, organizations used digital technologies for product development and the formation of innovative products via DI [35]. DI helps design innovative products, facilitates the introduction of novel, innovative products into the marketplace via emerging technologies, and leads to higher IP at the organizational level [36]. It used digital technology to describe the results and outcomes of IP [32]. DI transforms business-to-business operational activities and uses wider digital systems that help manage interactions through different actors in the network and achieve superior IP [9].

**Hypothesis 5.**  *Digital innovation is directly related to innovation performance.*

### 2.6. Digital Innovation as Mediator

Artificial intelligence and innovation performance link are mediated by Digital innovation. Enterprises are always looking for ways to use and apply AI knowledge to improve business operations through DI and increase their IP [37]. DI is becoming more popular in business firms because it is linked to AI and is used to increase intellectual property (IP) through new information and knowledge [38]. The ability of organizations to use and obtain AI resources and knowledge through DI can help their IP [4].Therefore, DI acts as a bridge between AI and IP and plays an important and valuable role for AI in increasing and improving the IP of firms. AI gives the organization infrastructure, a set of instructions, and a set of shared assumptions that serve as the basis for DI. It ensures that DI processes move forward, improving IP [39,40].AI is a firm’s dynamic ability that improves its ability to create innovative products in response to new DI technologies, ultimately increasing IP [41]. AI improves the learning mechanisms of firms so they can rapidly understand new DI technologies and acquire IP easily [42].

**Hypothesis 6.**  *The link between artificial intelligence and innovation performance is mediated by digital innovation.*

### 2.7. Mediationg Role of Digital Innovation

The IN assists organizations in acquiring additional information about ideas, innovative methods, and knowledge through DI in order to improve their IP [43]. It facilitates enterprises’ knowledge acquisition for the growth of innovative products, the quick implementation of the latest emerging technologies, and the recruitment of their resources to enhance IP [44]. INs aid in understanding how to enhance prospects for technological communications and thought collisions among businesses, which can greatly increase corporate progression and DI and finally result in developed IP [5]. The IN enables firms to continuously obtain technologically innovative knowledge and ideas through DI, combine this information with inside data, and generate numerous novel ideas and perceptions of product expansion in the firm to improve IP [7]. However, given that DIs serve as a bridge and means of communication between INs and IP, DI helps firms utilize accessible resources and adapt to fresh market trends to make new products and develop existing ones [5]. The IN provides a huge amount of practical and technical knowledge for the accomplishment of the DI in business to improve IP [27].

**Hypothesis 7.**  *Digital innovation mediates between innovation networks and innovation performance.*

**Figure 1:**
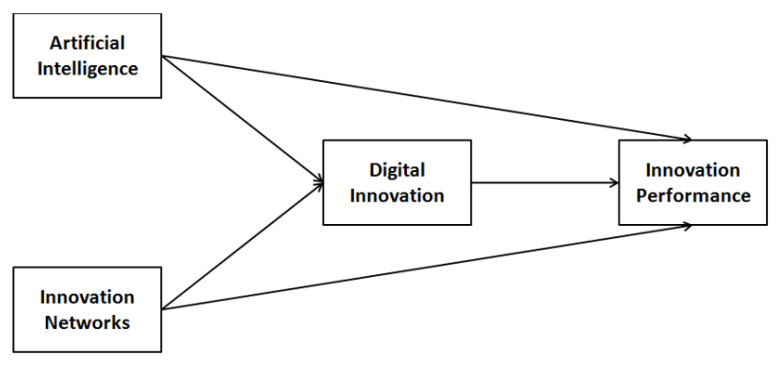
Theoretical Framework.

Based on the above discussion, the model that is tested by the current study is given in Figure 1.

## 3. Methodology

Methodology of the paper is discussed below:

### 3.1. Research Methods

In the current research, for data collection, quantitative research methods and random sampling techniques were used. The main strength of this study is that its findings should be generalized to the entire population based on a relatively small, representative sample.

### 3.2. Data Collection

Healthcare is an essential industry in any country. In the current research, a questionnaire was used as a survey tool for data collection from Chinese healthcare firms involved in developing the digital economy, such as their operating systems based on AI and DI’s latest technologies, and have the capacity of at least 250 beds. 

With the assistance of professional research assistants, data was collected by sending a questionnaire to respondents via postal mail, email, and other means. After five months of work, the research assistant received 376 responses from 985 questionnaires distributed via hard copy and electronically, of which only 340 were usable, resulting in a 68.5% return rate; the remaining 154 were incomplete and were not referred for further analysis. Before being distributed to respondents, the questionnaire was thoroughly reviewed by academics and experts to ensure that everything was clear.

### 3.3. Measures

The questionnaires were composed of two sections. The first section includes demographic variables such as hospital size, time period, respondent education, and experience. Section 2 of the questionnaire includes items to measure, i.e., AI, INs, IP, and DI. For all the variables in the current research, responses were recorded on a 5-point Likert-scale.

#### 3.3.1. Artificial Intelligence

AI was measured with a three-item scale, which is described in [45]. This construct describes how AI adoption helps in the attainment of targets, the combination of actors, and the performance of tasks for innovation practices in the era of intelligent machines.

#### 3.3.2. Network for Innovation

To measure the IN 5-item scale, adapted from [46], this variable describes how a firm uses its innovation network to identify systems or actions involved in encouraging innovation.

#### 3.3.3. Digital Innovation

DI is measured with three items prepared and used in previous research [47]. The example question is “Establishing advanced quality digital technologies in comparison to competitors”.

#### 3.3.4. Innovation Performance

IP refers to a company’s ability to develop improved, innovative products and new products for the company and the global market. It is measured by a five-item scale, adapted from previous studies [48]. The sample question is, “Our firm used the latest technologies and tools for product innovation”.

## 4. Analysis

### 4.1. Convergent and Discriminant Validity

Table 1 highlights the outcome of the discriminant and convergent validity [49]. Discriminant and convergent validities indicate that measurements were usable. All the results were acceptable, i.e., the value of FL is >0.70, and the AVE is >0.50. CR is >0.60, and the value of the Cronbach-alpha was greater than 0.70. Thus, all measurements, outcomes, and findings of the study were reliable and valid.

The Harman test was conducted to examine the CMB. This technique is used to identify common method bias (CMB). In the current research, all items of the constructs were loaded in exploratory factor analysis (EFA) to determine those aspects that are significant and responsible for variance, and their rotation was managed at zero level (Podsakoff et al., 2012), [50]. The findings show the presence of 18 distinct factors with an eight-value superiority over the one preceding factor. These 18 factors accounted for approximately 48 percent of the total variance, and the first factor accounted for 17 percent of the variance. Hence, CMV was not an issue, and no major CMB existed in the data.

### 4.2. Confirmatory Factor Analysis

Table 2 displays the CFA and model fitness results. The findings confirm that our 4-factor model is suitable for the data(χ^2^ = 1035.42, df = 470; χ^2^/df = 2.203; RMSEA = 0.06; CFI = 0.92; GFI = 0.91).

### 4.3. Correlation Result

Results depicted in Table 3 proved that Innovation Network (IN), Artificial Intelligence (AI), and DI were positively and significantly associated with IP (IP).IN is significantly affected by IP (r = 0.26 **, *p* value = Significant).AI and IP have a positive association (r = 0.38 **, *p* value = Significant). DI and IP have a considerable association (r = 0.32 **, *p* value < 0.001). The variance inflation factors scores corroborate that in current research, the issue of multicollinearity is absent as its significance was less than 10.0. Results depicted in Table 3 proved that the Innovation Network (IN), Artificial Intelligence (AI), and DI were positively and significantly associated with the IP (IP). IN is significantly affected by IP (r = 0.26 **, *p* value = Significant). AI and IP have a positive association (r = 0.38**, *p* value = Significant). DI and IP have a considerable association (r = 0.32 **, *p* value < 0.001). The variance inflation factor scores corroborate that in current research, the issue of multicollinearity is not present as its significance is less than 10.0.

### 4.4. Hypothesis Testing

SEM was utilized to check the study hypothesis. Table 4 presents that IN significantly predicts IP, and H1 was accepted (*β* = 0.26, *p* < 0.001). H2 shows that AI forecasts IP (*β* = 0.42, *p* < 0.001), and Hypothesis 2 was confirmed. H3 presents IN determines DI (*β* = 0.36, *p* < 0.001). Similarly, H4 suggests that A1 predicts DI(*β* = 0.28, *p* < 0.001). H5 shows DI predicts IP (*β* = 0.44, *p* < 0.001).

### 4.5. Mediating Effect of DI between AI and IP

Table 5 proposes that DI mediates between AI and IP. [51] analysis was used. Table 5 presents the results of the indirect effect of AI on IP through DI (IA→DI→IP). Path ‘a’ proposed AI that predicts DI (B = 0.442, t = 7.153, *p* < 0.000). Path ‘b’ proposes the direct effect of DI on IP (B = 0.286, t = 7.064, *p* < 0.000). Path ‘c’ proposes the total effect of AI on IP (B = 0.168, t = 3.557, *p* < 0.000). Path ‘c’ proposed that when DI was controlled, AI’s direct effect on IP was condensed and non-significant, indicating a mediation effect (B = 0.138, t = 0.079, *p* = 0.000). Path ‘ab’ shows the results of the indirect effect in the last row of Table 6. The indirect effect result shows that DI acts as a mediator (Beta = 0.267, Lower = 0.1270 to Upper = 0.2473 A normal test was conducted to examine the mediating effect of technological advancement. H6 was thus validated, and it is now established that DI serves as a mediator between the AI and IP links.

### 4.6. Mediating Effect of DI between IN and IP

H7 recommended that DI play a mediation role between IN and IP. Preacher and Hayes’ (2008) analysis was used. Table 6 presents the results of the indirect outcome of IN on IP through DI (IN→DI→IP). Path “a” is a proposed IN that predicts DI (B = 0.458, t = 7.413; *p* < 0.000). Path ‘b’ represented the DI’s direct influence on IP (B = 0.365, t = 7.088, *p*< 0.000). Path “c” proposed the entire impact of IN on the IP (B = 0.264, t = 3.820, *p* < 0.000). Path ‘c’ suggested that when DI was controlled, the direct outcome IN on IP was condensed and non-significant, indicating complete mediation (B = 0.127, t = 1.264, *p* = 0.131). Path ‘ab’ explains the results of the indirect effect in Table 5’s last row. The indirect effect results show that DI acts as a mediator (Beta = 0.184, Lower = 0.1040, and Upper = 0.2780). A normal test was also carried out to examine the mediation effect of technological progress. Therefore, H7 is established, as is the fact that DI mediates the connection between IN and IP.

## 5. Discussion

This study proposes seven hypotheses to investigate the effects of INs, AI, and DI on IP for the healthcare industry. H1 demonstrates that AI predicts innovation performance. The study’s findings support the notion that AI has a significant impact on IP. Our research is the first to present research that extends the brilliant harmony of AI beyond its simple linkage and proposes novel findings in terms of organizational performance and competitiveness. H1 findings reveal that AI helps in acquiring valuable information in the form of product information, forecasting and adopting market trends, and enhancing IP [13]. AI is the capability of computers to control robots to do jobs commonly done by intelligent beings [14]. H2 shows a direct association between AI and IP. The findings of H2 corroborate the positive linkage between IN and IP. The outcome demonstrates that INs make it simpler for firms to look for the necessary materials, data, and suggestions for boosting IP [17]. The utilization, absorption, and integration of knowledge into hospitals will be impacted by the structural qualities of an IN, which will then have an impact on the hospital’s IP [18]. H3 of our research shows that AI is associated with DI in the healthcare industry. According to the findings, AI contributes to DI by utilizing and implementing existing firm resources for the improvement of business operations in the healthcare industry. The H3 results show that AI enables the combination of physical and digital components to produce new market offerings, services, and products, generate new business models and processes, and increase the DI process for hospitals [2].

AI helps DI in hospitals by building, reconfiguring, and integrating external and internal skills to deal with a fast-changing environment [21]. Furthermore, H4 of this research presents a positive linkage between the IN and DI. The findings validate the statistically supported relationship between INs and DI. Previous research has also suggested that through DI; INs provide access to various novel information resources and capabilities, as well as interaction among artists, which boosts innovativeness in firms [31]. H5 of our study presents a positive relationship between DI and IN’s. The study about H5 showed that DI enables organizations to collect and share information and knowledge, improve IP, and adapt to new e-market trends [32]. It increases organizations’ internal innovation advantages through product improvement and new product development processes that lead to improved IP. DI can boost an organization’s competence in designing and developing new products and enhancing its IP [9]. Part 6 of our study proposes the mediating role of DI in the relationship between AI and IP.

These results showed that DI is a growing phenomenon for business firms due to its connection with AI in business concerns for increasing IP through new innovative knowledge and information [38]. Organizations’ capacity to use and acquire AI resources and knowledge linked with DI can positively influence firms’ (hospital)IP [4]. These findings indicated that DI is a growing phenomenon for business firms because of its relationship with AI in business concerns for increasing IP through new innovative knowledge and information [38]. The ability of organizations to use and acquire AI resources and knowledge related to DI can positively influence firms’ (hospital)IP [4]. Therefore, DI acts as a bridge between AI and IP and plays an important and valuable role for AI in increasing and improving the IP of firms. Hence, H6 of our study is supported. Finally, our research demonstrates how DI mediates the relationship between INs and IP. The findings indicate that the IN assists hospitals in acquiring additional information about ideas, innovative methods, and knowledge through DI in order to improve their IP [43]. It facilitates hospitals’ knowledge acquisition for the growth of innovative products, the quick implementation of the latest emerging technologies, and the recruitment of their resources to enhance IP [44]. In conclusion, the mediating role of DI has been proven through the findings of our study. From the findings of all previous studies, we recognized that they used different variables to partially examine their outcomes on IP and frequently used one variable that affected the innovation performance of an organization. Overall, this study model is comprehensive, unique, and adds to prior literature knowledge by investigating IN, AI adoption, and the role of DI in the achievement of innovation performance.

### 5.1. Theoretical Implications

This study supports the idea that DI has a significant role in determining IP for the healthcare sector in the context of AI and innovation networks. In the existing literature, only a few studies focused on how AI and INs enhance IP in the healthcare industry. This study looks into how AI and INs might boost the IP of the healthcare industry. Second, this study creates a framework for evaluating the performance of business innovation that shows how the interaction of several elements, such as AI, INs, and DI, can enhance the performance of business innovation in the healthcare sector. The third implication of the research at hand is the investigation of Ins and AI for enhancing DI in the healthcare sector. As a necessary condition for DI and IP, this research focuses on the gap between AI and IN’s. Fourth, existing literature explains that AI and INs are critical to firms’ innovation processes and performance; however, earlier studies have concentrated on how AI affects IP. In this study, we investigated and backed up the idea that DI has a mediating influence on the effectiveness of innovation. The importance of DI as an indicator of intellectual property has drawn the attention of many researchers. Digital innovation had a significant impact on IP as a result of AI and the IN. The current study supports the idea that DI serves as a mediator between AI, INs, and IP. Our study aims not only to illustrate how AI and INs affect IP but also to explain why DI and IP with AI and INs are enhanced. 

### 5.2. Practical Implications

This study makes some significant recommendations that hospital owners, practitioners, and hospital administration should put into practice. First, this study proposes that with the aid of IN abilities focused on mobilization and the integration of AI technology and human resources, the healthcare sector can enhance its innovation performance within the hospital. As a result, owners and senior management should concentrate on the development of innovation networks for improving hospital IP, allowing the general public to access state-of-the-art medical facilities. Second, the study contends that INs and AI are important pre-requisites and predictors of DI. In the healthcare sector, IN development and AI adoption have become significant potential factors to promote the critical competitive benefits of innovation performance. Therefore, hospitals must concentrate on AI development and enhance their IN, which in turn will raise DI and IP levels. Doing so will help them become more capable of achieving IP. Thirdly, to enhance the state-of-the-art facilities at hospitals, the government should ask hospital management and owners to integrate and improve their innovation network and implement advanced digital innovation practices in their daily operations to ultimately support the innovation performance of the healthcare industry. To increase innovation performance, innovation network implementation first encourages organizations to support digital innovation practices through AI adoption activities that protect and sustain innovation performance. Therefore, both government and enterprises at an organizational level can encourage innovative practices. Besides the economic support, they must promote innovative practices in enterprises by offering incentives in the form of subsidies, depreciation allowances, and tax exemptions.

### 5.3. Limitations and Future Research Directions

This paper’s theoretical and methodological restrictions offer opportunities for future study. Firstly, collecting cross-sectional data at different points in time might create a CMB issue; however, current research reveals that the innovation performance of the healthcare sector was determined through an innovation network, which can control the CMB impact. Therefore, future researchers must consider mixed method approaches to investigate the relationship between the innovation network and innovation performance results for a more inclusive analysis. Secondly, in this research, we considered digital innovation as a mediator between IN, AI adoption, and IP links. Although future research should use other psychological and social concepts, such as management trust, and work-life balance, moderated-mediation models must be investigated for testing the association between IN and IP, potentially offering a better understanding of their relationship. Thirdly, in this study, a quantitative research method was used for data collection; however, future studies should use a qualitative or longitudinal study method for data collection. Lastly, current research targeted CEOs, administration, and management; however, to gain a further comprehensive portrait of innovation performance at the organizational level, upcoming research should regard lower-level employees, including technical, HR, and personnel staff.

## 6. Conclusions

With the recognition of the significance of innovation performance, the healthcare sector realizes that innovation performance is linked with innovation network activities in innovation initiatives [52,53]. Particularly, this research tries to link nodes of the IN, AI adoption, and DI with innovation performance. Furthermore, current research has recognized an observable gap in existing literature knowledge on DI’s mediating role in a specified conceptual model. These research outcomes are congruent with practical recommendations about the linkage between IN and AI adoption practices and in what phenomena this relationship is strong. In conclusion, outcomes suggest that management, organizations, and policymakers should deem IN at the firm level by showing dynamic features that incentivize organizations to continue such practices.

## Figures and Tables

**Table 1:** Results of the Discriminant-Validity and FL.

Variables Description	F.L	T-Value	Alpha	C.R	AVE
Innovation Network			0.85	0.92	0.72
IN-1	0.72	15.77			
IN-2	0.83	14.55			
IN-3	0.85	13.56			
IN-4	0.74	15.47			
IN-5	0.76	14.22			
AI			0.87	0.94	0.74
A-Inti1	0.72	14.47			
AI-Inti2	0.85	15.22			
AI-Inti 3	0.86	14.66			
Digital Innovation			0.88	0.96	0.75
DI_1	0.82	15.63			
DI_2	0.85	14.56			
DI_3	0.83	15.77			
Innovation Performance			0.84	0.93	0.76
Inn Per-1	0.77	1563			
Inn Per-2	0.84	13.22			
Inn Per-3	0.85	14.55			
Inn Per-4	0.84	15.77			
Inn Per-5	0.86	15.77			

**Table 2:** Confirmatory Factor Analysis (CFA).

Model Description	χ^2^	Df	χ^2^/df	RMESA	GFI	CFI
Hypothesized-four-factor model	1035.42	470	2.203	0.05	0.93	0.91
Three factor model	1195.44	360	3.321	0.14	0.85	0.86
Two factor model	1247.55	385	3.240	0.16	0.73	0.74
Single factor model	1468.23	375	3.915	0.25	0.62	0.63

**Table 3:** Correlation, Standard Deviation and Mean.

Description		Average	S.D	Alpha	1	2	3	4	5	6	7	8
1	Hospital_Age	3.05	1.02	0.82	1.00							
2	Hospital_Size	1.34	0.44	0.86	0.116 **	1.00						
3	Respondent_Experience	1.78	0.32	0.85	0.215 **	0.86 *	1.00					
4	Respondent_Education	1.24	0.58	0.87	−0.03	0.07	1.00	1.00				
5	Innovation_Network	3.69	0.46	0.86	−0.02	−0.18	0.01	−0.10	1.00			
6	Artificial_Intelligence	3.58	0.47	0.85	0.04	−0.05	0.093 *	−0.02	0.164 **	1.00		
7	Digital_Innovation	3.79	0.63	0.82	−0.09	−0.15	−0.04	0.085 *	0.347 **	0.367 **	1.00	
8	Innovation Performance	0.45	0.49	0.84	0.03	−0.12	−0.05	−0.12	0.260 **	0.389 **	0.325 **	1.00

* = *p* < 0.005, ** = *p* < 0.001.

**Table 4:** Direct Hypothesis.

Model	Hypothesis Description	R2	F	B	T-Value	Sig.	Remarks
Model #1	Innovation Network→IP	0.178	14.085	0.267	4.720	0	H1 Approved
Model #2	AI→IP	0.324	62.170	0.428	7.250	0	H2 Approved
Model #3	IN→Digital Innovation	0.418	54.789	0.364	5.660	0	H3 Approved
Model #4	AI→Digital Innovation	0.367	71.885	0.286	7.560	0	H4 Approved
Model #5	DI→Innovation Performance	0.268	42.638	0.442	7.920	0	H5 Approved

**Table 5:** Indirect Effect of DI between AI and IP.

Description			Model Description	Beta	T-Value	SE	Remarks
**(Path a)→IV to Mediator**			**AI-DI**	0.442	7.153	0.042	0.000
(Path b)→Direct effect of mediator on DV			DI-IP	0.286	7.064	0.033	0.000
(Path c)→Total effect on IV on DV			AI-IP	0.168	3.557	0.054	0.000
(Path c’)→Direct effect of IV on DV			AI-IP	0.138	0.079	0.078	0.000
Model detail for DV Model: R^2^ = 0.5967; F = 531.2112; *p* = 0.000.							
**Bootstrap for indirect effect of IV on DV through mediator “ab path”**							
**Model Description**	**Data**	**Boot**	**Bias**	**SE**	**Lower**	**Upper**	**Sig.**
AI→DI→IP	0.267	0.328	0.004	0.328	0.1270	0.2473	0.0000

**Table 6:** Indirect Effect of DI between IN and IP.

Description			Model Description	Beta	T-Value	SE	Remarks
**(Path a)→I.V. to Mediator**			**IN→Digital Innovation**	0.458	7.413	0.053	Sig.
(Path b)→Direct effect of mediator on DV			DI→IP	0.368	7.088	0.043	Sig.
(Path c)→Total effect on IV on DV			IN→IP	0.264	3.820	0.067	Sig.
(Path c’)→Direct effect of IV on DV			IN→IP	0.128	1.269	0.089	Insig. (0.131)
Model Detail for Dependent Variable Model: R^2^ = 0.1422; F = 34.7321; *p* < 0.0001.							
**Bootstrap through mediator (Indirect effect) “ab path”**							
**Model Description**	**Data**	**Boot**	**Bias**	**S-E**	**Lower.**	**Upper**	**Remarks**
IN→DI→IP	0.184	0.16	0.002	0.43	0.1040	0.2780	Sig.

## Data Availability

Data is unavailable due to privacy or ethical restrictions.
